# Experiences From Older Patients Regarding Their Transition From the Acute Hospital to Their Home: A Phenomenological Study

**DOI:** 10.5334/ijic.9119

**Published:** 2026-04-20

**Authors:** Şirin Özkan, Elias De Coninck, Merel Leithaus, Geert Goderis, Hilde Verbeek, Mieke Deschodt

**Affiliations:** 1 Gerontology and Geriatrics, Department of Public Health and Primary Care, KU Leuven, Belgium; 2 Department of Medical Services and Techniques, Vocational School of Health Services, Bursa Uludag University, Bursa, Turkey; 3 Competence Center of Nursing, University Hospital Leuven, Belgium; 4 Institute for Research in Operative Medicine (IFOM), Witten/Herdecke University, Cologne, Germany; 5 Academic Center for General Practice, Department of Public Health and Primary Care, KU Leuven, Belgium; 6 Department of Health Services Research Care and Public Health Research Institute, Faculty of Health, Medicine and Life Sciences, Maastricht University, Maastricht, The Netherlands

**Keywords:** transitional care, older people, patient experiences, continuity of care, phenomenological study

## Abstract

**Introduction:**

Improving care transitions through better coordination and understanding patient experiences is essential for enhancing care outcomes. This study aims to explore the perceptions of older patients about their transition from hospital to home.

**Methods:**

A phenomenological design was used to explore the experiences of older adults aged 65 and older with at least one chronic condition. Participants (n = 16) were recruited from a geriatric department in Flanders, Belgium. Semi-structured interviews were conducted between 2020 and 2022, transcribed, and analyzed using thematic analysis in NVivo.

**Results:**

Three key themes emerged from the study: (1) adaptation to a new reality, where participants described difficulties in adjusting to new routines and navigating emotional and psychosocial changes; (2) emotional and self-management support, emphasizing the importance of assistance in maintaining independence and self-sufficiency while expressing concerns about becoming a burden to others; and (3) perceived quality of care, where participants expressed overall satisfaction with the care received but highlighted the need for clearer communication and more comprehensive information during the transition process.

**Conclusion:**

Older patients emphasize the need for independence and clear communication, calling for coordinated care that integrates their physical, emotional, and quality-of-care needs.

## Introduction

In recent decades, Europe has seen a significant rise of adults aged 65 and above [1]. As individuals age, they often experience an increased prevalence of comorbidities, which increases the demand for both formal and informal care services [2,3,4]. Because of the more complex health care needs, older adults also have a higher risk of hospitalization [5] and experience more frequent transitions between various care settings, such as hospital to home or long-term care facilities [6]. These transitions are frequently characterized by care fragmentation and poor coordination among healthcare providers, leading to interruptions in continuity and diminished quality of care. Such disruptions can complicate efforts to address the increasingly complex needs of elder adults [7,8].

One of the most critical transitions is from hospital to home, during which nearly one in five patients experience an adverse event, 62% of which are preventable [9]. This risk is especially high for older adults, who are particularly vulnerable due to their complex medical and social needs [10]. In fact, the rate of adverse events during this transition is twice as high as what patients typically face while being hospitalized [11,12,13]. As a result, patients face an increased risk of hospital readmission, which not only prolongs their overall care but also increases their exposure to hospital-associated risks, such as infections [14]. The risks associated with inadequate care transitions are substantial, as suboptimal handoffs between care settings directly contribute to higher readmission rates. These frequent readmissions undermine the intended advantages of shorter hospital stays [15].

Care transitions also affect the daily lives of older adults and their informal caregivers. These periods are often marked by confusion, feelings of powerlessness, and a loss of autonomy, particularly when decisions are made on behalf of the older person without their active involvement [16,17,18,19] further complicating the transition process.

Given these challenges, improving care transitions in older adults is essential for enhancing patient outcomes and their overall quality of life. Nevertheless, progress is often hindered by a by a limited understanding of patient’s experiences during this crucial time [20]. Older adults offer a valuable perspective on both strenghts and shortcomings of transitional care, providing essential insights for identifying areas that need improvement [21]. Exploring their experiences can inform strategies to better coordinate care, enhance communication among healthcare providers, and address the emotional and psychological challenges associated with transitions [19]. The aim of this study is to explore and understand the perceptions of older patients regarding their transition from hospital to home and the continuation of care in the home setting.

## Methods

### Study Design

This qualitative study used a phenomenological design to understand the perspectives of participants and interpret the meaning. This method was used to describe and interpret the lived experiences of older patients during the transition period, as it seeks to uncover the deeper meanings behind everyday experiences by interpreting them during the early post-discharge phase, i.e., within two weeks after hospital discharge, and by revealing the subjective meanings embedded in their narratives [22]. This design is consistent with the study’s objective of capturing the emotional and relational dimensions of care transitions.

### Participants

We included patients aged 65 years and older with at least one chronic disease who were hospitalized in the geriatric department of the University Hospital Leuven, a large tertiary academic hopsital in Belgium. Eligible participants were Dutch-speaking and residing either at home or in a service flat and returning there at the time of the interview. Participants with a confirmed diagnosis of significant cognitive impairment such as dementia, delirium, or psychosis, were excluded from the study. Individuals with significant hearing or speech impairments or residing in a residential care center were also excluded.

### Recruitment procedure

Participants were recruited from three geriatric wards at the study hospital. To minimize any risk of perceived coercion, clinical nurses were involved only in identifying eligible participants and did not take part in the recruitment process. The researchers responsible for recruitment played no role in clinical care, and all interviews were conducted after discharge to avoid any perception that study participation might affect clinical care. The researchers provided each eligible participant with detailed verbal information regarding the study during their hospital stay, emphasizing that participation was entirely voluntary and would not affect their care in any way. Participants were given time to consider their decision and ask questions before providing written informed consent. Consent also included permission for others—such as family caregivers—to be present during the interview, if requested by the participant. Participants who agreed to take part in the study were given informed consent forms. Once they provided written consent, face-to-face interviews were conducted with them in their homes. The study was approved by the Research Ethics Committee UZ/KU Leuven (file numbers MP012112 and MP017276).

### Data collection

Data were collected through individual semi-structured interviews by one of two interviewers. All interviews were conducted face-to-face in participants’ homes to ensure a comfortable and neutral setting. The interview questions were informed by key themes identified in previous studies which included experiences during the hospital stay, preparation for discharge, the discharge process itself, arrival home and post-discharge adjustment These themes reflect important stages of transitional care and informed the development of the interview schedule. Interviews were conducted within two weeks after hospital discharge, which is a critical period when patients adapt to new care routines at home and which is in line with previous studies [23,24]. In some instances, caregivers were present with permission of the participant, while others were conducted privately with the older adults [25,26]. Data collection occurred between January 2020 and September 2022 with temporary pauses due to staff absences and COVID-19 measures on the participating wards. The total data collection period was 10 months.

### Data analysis

Data analysis was conducted following the Leuven Qualitative Analysis Guide (QUAGOL) framework [27]. All audio-recorded interviews were transcribed verbatim into Word documents capturing the interviewees’ tone and pauses, with participant confidentiality ensured through pseudonymisation. In case a caregiver was present, only the participant’s statements were analyzed. The research team began by repeatedly reading the transcripts (Stage 1: Re-reading the interviews) to gain an understanding of each participant’s narrative. This was followed by the development of narrative interview reports (Stage 2), which captured the essence of each story. From these reports, a conceptual interview scheme (Stage 3) was constructed to identify emerging ideas and relational patterns. To ensure the relevance and consistency of this scheme, a fitting test (stage 4) was conducted across the interviews. A constant comparison process (Stage 5) was then applied to refine and align concepts across cases. The team then drew up a list of concepts (Stage 6) to guide the coding process (Stage 7), returning to the raw data to ensure a grounded interpretation. These codes were analysed (Stage 8) to uncover deeper meanings, leading to the extraction of the essential structure (Stage 9) of the participants’ experiences. Finally, the findings were synthesised into a coherent description of the results (Stage 10) and organised into sub-themes and overarching themes using NVivo 14 to support thematic analysis [28]. To further illustrate the findings, various visual representations, including charts, diagrams, and maps, were created. The paper was written according to the Consolidated Criteria for Reporting Qualitative Research (COREQ) guidelines [26,29].

## Results

### Participants

A total of 16 interviews were conducted with older people who transferred from hospital to home. Participants had an average age of 82.6 years (range: 75–94 years) and eight (50%) were female. Detailed information regarding the participants’ demographics is provided in Table 1.

**Table 1:** Demographic information of participants.


	DATA COLLECTION WAVE	LENGHT OF HOSPITAL STAY	GENDER	AGE	LIVING STATUS

1	1	3 weeks	M	94	Alone

2	1	1 week	M	75	With partner

3	1	5 weeks	M	85	With partner

4	1	1 week	M	76	With partner

5	2	1 week	M	75	With partner

6	2	2 weeks	M	78	Alone

7	2	2 weeks	F	87	Alone

8	2	10 days	M	81	With family member (son)

9	2	1 week	F	91	Alone

10	2	2 weeks	F	70	Monastry

11	2	3 weeks	M	80	Alone

12	3	10 days	F	99	With family member (daughter)

13	3	4 days	F	91	Alone

14	3	1.5 week	F	81	Alone

15	3	2 weeks	F	86	Alone

16	3	4 weeks	F	83	With partner


### Results from the interviews

Three core themes emerged from participants’ experiences with transitional care: 1. Adaptation and adjustments at home and new reality, 2. Need for emotional, psychological, independence and self-management support, and 3. Perceived quality of care (Table 2).

**Table 2:** Thematic mapping and the common core of experiences.


COMMON THEMES	SUB-THEMES	SPECIFIC TOPICS

**1. Adaptation to new reality**	1.1. Adjustment to new routines	Adjustment physical limitation Adjustment nursing, physiotherapy services and safety measures at home

	1.2 Emotional and psychosocial adapation	Acceptance of aging and decreasing independence Social support from informal caregivers

**2. Emotional and self-management support**	2.1. Support for maintaining independence and self-sufficiency	Strong desire for independence Appreciating and accepting the support of family members

	2.2. Concern to be burden	Concerns regarding future care being burden to family members

**3. Perceived quality of care**	3.1. Improving communication and coordination between hospital discharge and primary care integration	Inadequate information provided about discharge and post-discharge instructions. Need help making arrangements during and after discharge

	3.2. Medication management	Unclear information regarding medication Need for more comprehensive information related to medication and follow-up care at home

	3.3. Satisfaction with health services	Despite overall satisfaction from hospital stay a strong desire to return home Overall satisfaction with home care services, along occasional difficulties with staff availability and performance


#### Theme 1: Adaptation to new reality

Transitioning from hospital to home represents a critical milestone in a patient’s recovery journey. This process entails adjustments necessary to ensure a smooth and safe reintegration into a familiar environment. The primary theme that emerged from the interviews centers on the physical and psychological adjustments required for older adults as they establish new routines. Following the transition, participants reported significant challenges in adapting to changes in their lives, particularly concerning their healthcare needs and the modifications they made to their homes and daily routines.

##### 1.1. Adjustment to new routines

Participants indicated that they had made various modifications to their living spaces and daily activities to better accommodate their evolving health needs. This involved integrating new activities and schedules designed to manage their health and well-being. Participants reported adopting post-hospitalization routines that included an increased reliance on mobility aids and expressed that even simple tasks, such as walking felt burdensome. Also, changes in medication therapy, such as adjustments to their insulin regimen, were also noted as significant factors in their adaptation process. Additionally, participants noted the frequency of assistance received from healthcare providers for essential tasks, including bathing, dressing, and medication administration, further illustrating the adjustments necessary for their ongoing recovery.

“I have to be more careful, I feel like I need to use my cane more often than before because I have to watch out. I already had balance issues, and it seems to have gotten worse.” (Female; 91 years)

“Here [at home] I walk around more often and that is already tiring … Here I walk around a bit more and with more walking around, yes I have more pain. … I can’t eat well.” (Female; 99 years)

The organisation of nursing and physiotherapy services at home plays a crucial role in ensuring continuity of care. This support is vital as older adults manage their daily activities such as cooking and personal care. Participants noted that these services were crucial for helping them adjust to new situations and providing important support during this transition.

“I take care of my own cooking, bathing and laundry, I do what I can myself, my social assistant helps me with everything else.” (Male; 78 years)

“Actually, but now it will be when the physiotherapist comes. That’s not every day, it’s every two days he comes. … this morning they washed my head… Tomorrow they will come and do my back, and I will do the rest” (Male; 81 years)

Older patients also emphasised the adaptation of safety measures at home by wearing an alarm bracelet connected to a centralised monitoring system. Such devices provide reassurance for both the older person and their family, guaranteeing their safety and well-being in the home environment.

“If things didn’t go well, my daughter would sleep here, but when I feel better, I’d prefer for her to sleep at home. I can press a button on my wristband, and she’ll be notified and come over if needed.” (Female; 99 years)

Despite significant health challenges and lifestyle changes, resilience and adaptability are demonstrated. Activities such as reading and completing crossword puzzles are engaged in to maintain mental activity and a sense of normalcy in daily life.

“I can’t just sit around doing nothing. This is my fourth book of crosswords.” (Male; 94 years)

##### 1.2. Emotional and psychosocial adapation

Throughout the interviews, participants’ emotions ranged from feelings of powerlessness, anxiety to acceptance, gratitude and reliance. Besides the physical adaptation, participants also refer to the process of psychological adaptations to accept to their new reality. This psychological acceptance is reflected in their willingness to adapt to new routines and rely on external help, especially from health professionals and family members.

“Now the paper[medication list] is here, and my daughter has simplified it a bit so I could write down ‘morning’ and ‘evening’ and put it in a box, allowing me to prepare it myself, since all my medications have changed. What I used to do on my own, I still do now, but with the help of that paper.” (Female; 99 years)

Participants emphasized the anxiety associated with sudden transitions and the necessity of to adapting to new limitations, such as reliance on family assistance and home care services.

“They didn’t give any information beforehand, they gave that the day before [discharge] around 4 o’clock because visiting hours were from 3 o’clock, yes, around 4 o’clock they came to say that you could go home the next day.” (Female; 81 years)

“I was given that note [prescription and instructions for physio] and my daughter arranged that herself. Yes, I think she did that, yes, she gave the note to the physiotherapist.” (Female; 91 years)

A substantial reliance on caregivers and healthcare professionals has become an integral part of the participants’ daily lives. This dependence represents both a practical adaptation and a crucial psychological adjustment to their new reality.

“The ones who help me put on my compression socks come everyday morning and every evening, also on saturday and sunday.” (Female; 86 years)

“I do get good help, people who used to work in my school and who come to help me a lot.” (Male; 94 years)

Participants expressed a strong desire to maintain their independence, despite struggling to emotionally accept the realities of ageing. They showed an ability to adapt to their current circumstances. Their reflections on health and aging offer important insights into how they view their present situation.

“In a few days I will be 76, but I don’t feel like an old person I can’t explain that maybe. I am old, but I don’t feel that. I don’t think I ever will. …” (Male; 76 years)

#### Theme 2: Emotional and self-management support

Although older adults often demonstrate independence and resilience during transitions, they experience emotional challenges due to post limitations, particulary concerning physical discomfort and a percieved reduction in autonomy. Participants reported difficulties in accepting these changes; however, they placed significant value on maintaining their independence and autonomy in healthcare decisions. They expressed a strong desire to actively participate in choices related to discharge planning and medication management.

Family members play a crucial role in supporting older adults throughout their healthcare journey, advocating for their needs during hospitalization and coordinating care following discharge. Furthermore, healthcare providers are expected to engage in proactive communication to ensure continuity of routines during transitional care, highlighting the importance of open dialogue and access to information for both patients and their families.

##### 2.1. Support of being independent and self-management

The participants demonstrated a sense of independence and resilience, despite facing health challenges and advanced age. They placed a high value on their autonomy in making decisions about their healthcare. They expressed a strong desire to remain active and engaged in life. Alongside this desire for independence they expect that their limitations will be recognized, respected, and supported by their care network.

“I inject myself. The nurse should not do that. I have been injecting myself for how many years, since 1993 I am injecting myself.” (Female; 87 years)

“I can take care of myself because I have always done that. I spent three years with a sick wife.” (Male;78 years)

The role of family members in providing support and companionship is highlighted as essential. While older adults greatly value their independence, they also recognize and appreciate the emotional and practical assistance provided by family members.

“I wish that my sons would come more often, it’s like they are scared. I wouldn’t go running, because I am not able to [laughs]. I always think I’d better leave this world, it would be better for them, and I wouldn’t have to suffer.” (Female; 81 years)

“I don’t like to depend on another person, I will say it as it is. I try to take care of myself, that I don’t have to bother other people”(Female; 86 years)

Participants frequently expressed gratitude for the support provided by friends and family, who played a significant role in facilitating the return home. Family and friends were actively involved in ensuring the well-being of older adults, taking initiatives such as inquiring, about discharge dates and coordinating post-discharge care arrangements. Their involvement was highlighted as essential for ensuring a smooth transition to home care and for providing ongoing support during the recovery process.

“For older people, it’s actually not always easy. Right now, my family is still here… But, and imagine you have children who don’t look after you… That you are alone, what then? And there’s nothing for eh… and that’s bad.” (Male; 75 years)

“That brings joy, because when I came back from the hospital, the neighbours brought me a small vase with flowers and a candle. I thought that was amazing, that doesn’t need to be big. It’s the thoughtfulness that counts.” (Female; 86 years)

##### 2.2. Concern of being a burden

Some participants expressed concerns regarding future care, primarily driven by the fear of losing the ability to manage their health and becoming a burden to their family members. They raised concerns about the potential loss of autonomy associated with transitioning to a nursing home, where the fear of being treated like a child and losing control over one’s environment emerged as a significant emotional burden. This highlights the critical importance of preserving dignity and autonomy in older person’s care. While older adults seek companionship and assistance, they simultaneously fear becoming a burden on their families and are apprehensive about the financial implications of long-term care.

“I don’t like being a burden to someone, I never have been. … The doctor also said “you’d better be home”. [She doesn’t want to go to nursing home] No, I am not selling my house, I had to work hard for that.” (Female; 81 years)

“If you can’t sleep, you go for a walk, but I had to stay in my room. I didn’t like having the door shut, there [in the hospital] the door had to be shut. I preferred the door open, but that wasn’t allowed. It had to be closed. … I had to listen. You are a child. With our own children too, we have to listen to them now, we have nothing to say here either.” (Female; 83 years)

#### Theme 3: Perceived quality of care

While participants expressed generally satisfaction with the care they received, they identified specific areas for improvement, particularly in sufficient information during the discharge, medication management and coordination among healthcare providers.

##### 3.1. Improving communication and coordination between hospital discharge and primary care integration

Participants generally expressed satisfaction with the care they received but highlighted critical areas for improvement, particularly in communication and coordination between hospitals and primary care providers. Recurring issues included unclear receiving limited discharge information, medication management, need to arrange care details themselves and fragmented communication among healthcare professionals, patients, and families. These concerns outlines the need for enhanced collaboration across healthcare settings to ensure continuity of care and better patient outcomes

Participants expressed a desire for improved communication and explanation from healthcare providers, particularly regarding their condition and care plan. They pointed out instances where they felt information was insufficient or inadequately provided regarding discharge and post-discharge instructions.

“They [health professionals] didn’t tell me much, just that I was allowed to go home on Saturday. That’s all they said” (Male; 75 years)

“Actually, the communication is not that good, there is not much communication, they [health professionals] don’t tell you much” (Male; 81 years)

Some older individuals reported that they or their relatives had to personally arrange for a home nurse to visit regularly after discharge from the hospital or to ensure follow-up care by their general practitioner (GP). This situation poses significant challenges for older adults who lack family support or are physically unable to manage these arrangements themselves.

“My cousin informed the home nurse about my return home” (Female; 91 years)

“After three weeks in the clinic, the first thing I did was to give away my car because I could no longer drive. Then I immediately called for family help because I could no longer cook. I arranged everything myself. “ (Male; 94 years)

##### 3.2. Medication management

Participants commonly expressed a need for more comprehensive information regarding medication and follow-up care at home. They found the written materials provided about post-discharge care and medication lists to be insufficient, and preferred for verbal clarification of these instructions. During this time, family members frequently took on the responsibility of helping to clarify things that older people did not understand. This underlines the recurring importance of the family in facilitating effective communication and contributing to decision-making throughout the care process.

“They [health professionals] didn’t tell me much, that I could go home on Saturday, that’s all they said. The medication and how to take it was printed on paper. But those were just the drugs I was already taking before.” (Male; 75 years)

“I started asking “yes but when can I actually go home?” So, I asked it Friday the first week …. they gave more information to him [family member], information about taking medication and everything, than they gave to me.” (Female; 99 years)

Frequent changes in medication regimens, combined with insufficient information, can increase anxiety among older adults. In such situations, general practitioners (GPs) are viewed as the most trusted source for ensuring consistent medications management. Participants often expressed satisfaction with regular visits and medical care from their GP, highlighting the trust in the doctor-patient relationship. They also emphasized the need for improved coordination and communication between the hospital and the GP.

“Medication is really a problem. Every time I go to the hospital, it changes. Then something is stopped, or something is added and when I go to my GP, sometimes it changes back…. My GP is allowed to know everything that happens to me, and all information is sent to him. However, sometimes the hospital hasn’t sent details about my treatment yet. When I ask about it, he checks his computer and confirms it hasn’t arrived yet, but reassures me that it will soon.” (Male; 94 years)

“If I had a problem now, I would call my GP, who is already aware of everything since I have already had a visit.” (Male; 75 years)

##### 3.3. Satisfaction with health services

The participants expressed overall satisfaction with the care they received during their hospital stay, highlighting the timely and attentive nature of the care and commending the professionalism of the hospital staff. However, despite this satisfaction, they conveyed a strong desire to return home, emphasizing the significance of the familiarity and comfort associated with the home environment.

“I really can’t complain about the hospitalisation, they did their best. I am satisfied. They are working on it; they are concerned, and they won’t let you down easily I would say.” (Male; 70 years)

“When I had more, I would rather be there [in the hospital] but when it got better, not anymore. I was very happy the day I went home.” (Female; 81 years)

The quality of home care services was generally viewed positively, with participants expressing overall satisfaction. However, they reported experiencing both positive interactions and occasional challenges, particularly concerning staff availability and performance.

“There are people who work hard… and there are people who don’t do much… They are really working for you, the only thing is that sometimes you have to wait for a long time. Yes, that’s true… but they can’t help it. And the nurses as well on weekends they have to work with fewer people and then you have to wait.” (Male; 94 years)

**Figure 1:**
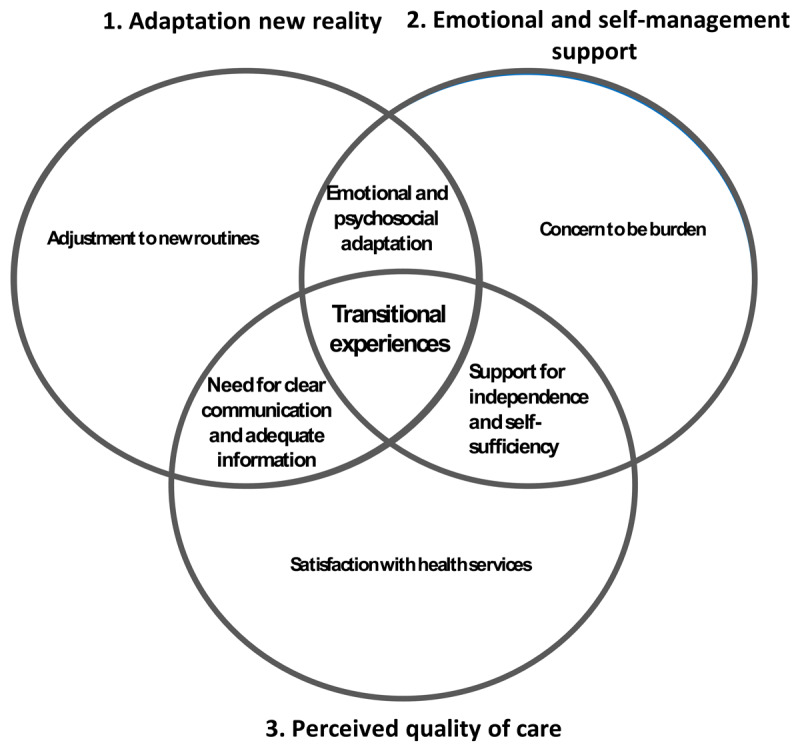
Schematic overview of the identified core themes and subthemes and how they relate to each other.

These themes are deeply interconnected, reflecting the comprehensive experience of participants during their transition from hospital to home. Figure 1 illustrates how each theme is deeply intertwined. Adaptation (Theme 1) lies at the heart of the transition process of older adults, and the emotional and self-management support (Theme 2) they receive influences the smoothness of this transition. The perceived quality of care (Theme 3) from healthcare providers, particulary in terms of communication and coordination, acts as a foundation that either strengthens or hinders their ability to adapt and maintain independence. Emotional well-being and independence are difficult to maintain without proper support and clear communication from healthcare providers, which in turn impacts how effectively older adults can adjust to their new routines at home. Ultimately, the quality of care influences both the adaptation process and the emotional resilience required during this transition.

## Discussion

This study shows that the transition from hospital to home poses significant physical and psychological challenges for older adults, evidenced by their need to adapt their living environments, routines, and daily activities to accomodate new health requirements. This transition is often accompanied by emotional impacts, including uncertainty and regarding the management of their care in a home setting. However, participants reported that the support received from nursing care and physiotherapy services at home reduced these concerns. Consistent with findings from Brooks et al., the customization of home care therapies to match patients’ lifestyles and environments was shown to alleviate feelings of uncertainty and foster a greater sense of comfort in navigating their post-discharge care [30]. This adjustment process also involves resilience and adaptability, as older patients gradually learn to manage self-care tasks. According to Allen et al., when participants in post-transition care developed self-care skills, including managing complex medication regimens, colostomies, urinary catheters, and blood sugar measurements, their experiences contributed positively to both their independence and self-efficacy [31].

Our findings emphasize the need for emotional and psychological support during the transition from hospital to home. During these care transitions, older adults often face emotional challenges that are intensified by the sudden loss of independence and physical discomfort. Many older adults, having been self-sufficient for much of their lives, struggle with the fact that they become dependent of others, as they wish to maintain control over their care and take their own decisions. Support from family and healthcare professionals was perceived as highly valuable. Consistent with other studies, older adults find adjusting to new routines and physical limitations emotionally challenging as they adapt to slower paces and increased reliance on caregivers. Family support is crucial not only for daily care but also for building emotional resilience and self-management skills [31]. Families particularly aid the transition by helping with care decisions and arranging home services, easing the re-establishment of familiar routines [32]. This perspective can be enhanced by a “caring societies” approach, which broadens care as a shared responsibility among governments, communities and the private sector, not just families or individuals. Though challenging, this perspective emphasizes the emotional and relational benefits of care, promoting a more compassionate and connected society [33].

Participants also expressed anxiety about the sudden transition to their home. Similarly, Brooks et al., [30]. found that older adults often felt uncertain about whether the care they receive adequately supports their unique needs in their home environment, leaving them feeling unprepared and worried about future care. Effective communication with healthcare providers is crucial alleviating anxiety, ensuring that older adults feel safe and informed about their treatments [34]. When transitioning home, the consistency and care provided by healthcare professionals enhance older individuals’ confidence in managing their medications independently, reinforcing their sense of control and independence. Studies suggest that involving patients in medication and care decisions is fundamental to fostering this autonomy, as it empowers them to feel more prepared and capable in managing their health [34]. In addition, encouraging patient empowerment by involving patients in managing their follow-up appointments, medications, and health goals has been shown to facilitate a smoother transition home [35]. Practical tools for managing post-discharge care, such as booklets, record sheets, and simplified discharge letters, have been shown to enhance patient engagement and can help older adults retain independence and better manage the emotional and practical challenges of returning home [36]. In addition, video-assisted and digital discharge education systems hold considerable potential to enhance post-discharge care; however, their effectiveness depends on factors such as patients’ digital literacy, the clarity and personalization of the content, and the quality of the implementation process [37].

Participants frequently emphasized the need for more detailed and comprehensive information about managing medications at home. Many older adults struggle to understand why their medication regimens are altered and may hesitate to voice their concerns during discharge, which can lead to confusion and difficulties in managing their post-discharge [34]. Nelson and Carrington proposed integrating communication tools with electronic health records to enhance medication reconciliation and clarify changes in patient prescriptions across different care settings [38]. Furthermore, Kraun et al., highlight the importance of providing comprehensive information about follow-up care, noting that older adults rely on timely, accessible, and clear instructions [32]. Unfortunately, many patients feel overwhelmed by information delivered in the hospital, often while they are sedated, fatigued or preoccupied, making it difficult to retain critical details. Therefore, opportunities for patients to ask questions and clarify their care instructions before and after returning home are invaluable [30].

In this study, the quality of both hospital and home care services was generally perceived positively, with participants expressing overall satisfaction. However, some challenges were noted regarding, enhanced collaboration across healthcare settings, staff availability and performance. Consistent with findings from other studies, older adults expressed a strong desire to regain their independence and return to the familiarity of their home environment, even when they reported satisfaction with the care services provided [31]. The three identified main themes represent the dynamic and interconnected relationship between these themes. They highlight how physical adjustments, emotional wellbeing, and the quality of healthcare are not isolated issues but parts of a broader, holistic experience that impacts older adults’ recovery and quality of life.

### Limitations

This study has certain limitations. First, not all interviews reached the anticipated depth of insight. While all participants were able to articulate their experiences, many encountered difficulty fully expressing their thoughts, feelings, and especially their specific needs, as has been reported as a limitation in interviewing frail older adults [39,40]. This limited the study’s ability to comprehensively capture and describe the emotional experiences of older adults, making it challenging to characterize an overall emotional state. Second, data were gathered exclusively from older individuals discharged from a single hospital. Third, the data collection occurred in three separate rounds due to the COVID-19 pandemic, which may have introduced variability in participants’ assessments over time. Changes in discharge routines and care practices during these intervals could have influenced participants’ experiences of transitional care, potentially affecting the study’s findings. Consequently, given the potential impact of perceived healthcare service quality on the transition process, this single-hospital sample restricts the generalizability of findings to a broader population.

### Implications for future care and research

The findings highlight several implications for improving hospital-to-home transitions for older adults requires a focus on family-centered care, enhanced communication, and support for patient autonomy. First, family involvement plays a crucial role in reducing transition-related anxiety, highlighting the need for healthcare professionals to actively engage family members in discharge planning and care coordination. Structured programs that facilitate family involvement using face-to-face or online tools could be studied to assess their effects on patient outcomes and emotional well-being. Secondly, the study suggests a need for improved communication and collaboration between hospital staff, home care providers, and family members. Future research could focus on developing and testing digital tools or educational interventions, such as user-friendly health portals or interactive guides, that promote seamless communication and information sharing across care settings. These tools could help older adults better understand and manage their care, increasing their autonomy and self-efficacy. Additionally, future care practices should balance support with respect for independence, with programs designed to promote self-management skills and independence among older adults, providing insights into best practices for safe, respectful care.

## Conclusions

The study highlights the patient journey from hospitalization to transitioning to home care, emphasizing both practical and emotional aspects of this experience. The frequent assistance from healthcare providers and home care staff underline the essential role these services play in supporting physical recovery and facilitating a smoother adjustment to new routines. Family involvement also proved valuable, helping to reduce anxiety associated with sudden lifestyle changes by providing emotional support and actively coordinating post-discharge care. Throughout this process, older adults expressed a strong desire for independence, highlighting the critical need for clear communication regarding treatment plans and follow-up care. This emphasis can be interpreted as a call for coordinated support across care settings, ensuring smooth transitions and collaboration among healthcare providers. Such efforts aim to meet the needs, preferences, and circumstances of older adults by integrating physical adaptation, emotional resilience, and perceived quality of care.
